# PredPRBA: Prediction of Protein-RNA Binding Affinity Using Gradient Boosted Regression Trees

**DOI:** 10.3389/fgene.2019.00637

**Published:** 2019-08-02

**Authors:** Lei Deng, Wenyi Yang, Hui Liu

**Affiliations:** ^1^School of Computer Science and Engineering, Central South University, Changsha, China; ^2^School of Software, Xinjiang University, Urumqi, China; ^3^Lab of Information Management, Changzhou University, Changzhou, China

**Keywords:** protein-RNA interactions, computational approaches, binding affinity, gradient boosted regression tree, sequence and structural features

## Abstract

Protein-RNA interactions play essential roles in many biological aspects. Quantifying the binding affinity of protein-RNA complexes is helpful to the understanding of protein-RNA recognition mechanisms and identification of strong binding partners. Due to experimentally measured protein-RNA binding affinity data available is still limited to date, there is a pressing demand for accurate and reliable computational approaches. In this paper, we propose a computational approach, PredPRBA, which can effectively predict protein-RNA binding affinity using gradient boosted regression trees. We build a dataset of protein-RNA binding affinity that includes 103 protein-RNA complex structures manually collected from related literature. Then, we generate 37 kinds of sequence and structural features and explore the relationship between the features and protein-RNA binding affinity. We find that the binding affinity mainly depends on the structure of RNA molecules. According to the type of RNA associated with proteins composed of the protein-RNA complex, we split the 103 protein-RNA complexes into six categories. For each category, we build a gradient boosted regression tree (GBRT) model based on the generated features. We perform a comprehensive evaluation for the proposed method on the binding affinity dataset using leave-one-out cross-validation. We show that PredPRBA achieves correlations ranging from 0.723 to 0.897 among six categories, which is significantly better than other typical regression methods and the pioneer protein-RNA binding affinity predictor SPOT-Seq-RNA. In addition, a user-friendly web server has been developed to predict the binding affinity of protein-RNA complexes. The PredPRBA webserver is freely available at http://PredPRBA.denglab.org/.

## Introduction

Protein-RNA interactions play a crucial role in many biological processes, such as gene expression and its regulation (Keene, 2007; Glisovic et al., 2008). To understand the mechanisms of these biological processes, the three-dimensional atomic structure of proteins and RNAs in bound and unbound conformations is essential. However, dissecting the 3D structure of protein-RNA complexes by X-ray crystallography and nuclear magnetic resonance spectroscopy is difficult and slow to date, due to the flexibility of the interacting partners of protein-RNA complexes.

In the past decade, many methods have been developed to identify protein-RNA interactions *via* experimental technique (Hafner et al., 2010) and computational prediction (Kim et al., 2006; Zhao et al., 2010; Fernandez et al., 2011; Dror et al., 2012; Liu and Miao, 2016). Setny and Zacharias (2011) developed a coarse-grained force field for protein-RNA docking and identified one of seven unbound protein-RNA cases from top 100 predicted samples. Tuszynska and Bujnicki (2011) published two knowledge-based scoring functions that were tested on eight unbound protein-RNA docking baits produced by the GRAMM program. Their results showed that these potentials were identified near the natural structure in four of the eight samples. Meanwhile, Li et al. (2012) raised a question about the propensity of residues-nucleotides, and they found that the secondary structure of RNA plays a crucial role in predicting residue nucleotide propensity potential. To evaluate the performance of these computational methods, Barik et al. published a protein-RNA docking benchmark (Barik and Bahadur, 2012), which significantly increased the number of experimentally determined protein-RNA complex structures and their unbound structures in the Protein Data Bank (PDB) (Berman et al., 2000). The protein-RNA docking benchmark dataset has been widely used to develop computational methods for studying protein-RNA interactions, including docking (Guilhot-Gaudeffroy et al., 2014; Guo et al., 2013; Iwakiri et al., 2016) and knowledge-based scoring functions (Huang and Zou, 2014; Yan and Wang, 2013) for the prediction of RNA binding sites in protein structures (Miao and Westhof, 2015), role of water molecules at the protein-RNA interface (Barik and Bahadur, 2014), and discovery of binding hotspots at the protein-RNA interface (Barik et al., 2015).

Although the protein-RNA docking benchmark has played an important role in studying multiple aspects of protein-RNA interactions, it is still somewhat inefficient in quantifying the binding affinity of proteins-RNA interaction. The standard non-redundant dataset of protein-RNA complexes is a prerequisite for the development and validation of protein-RNA binding affinity studies. Since lack of protein-RNA binding affinity data sets has become a bottleneck in the development of more accurate scoring functions, Yang et al. (2013) developed a dataset of protein-RNA binding affinity in 2013, which includes the quantitative binding affinities of 73 protein-RNA complexes. However, few methods for predicting the binding affinity of protein-RNA complexes have been developed.

**Figure 1:**
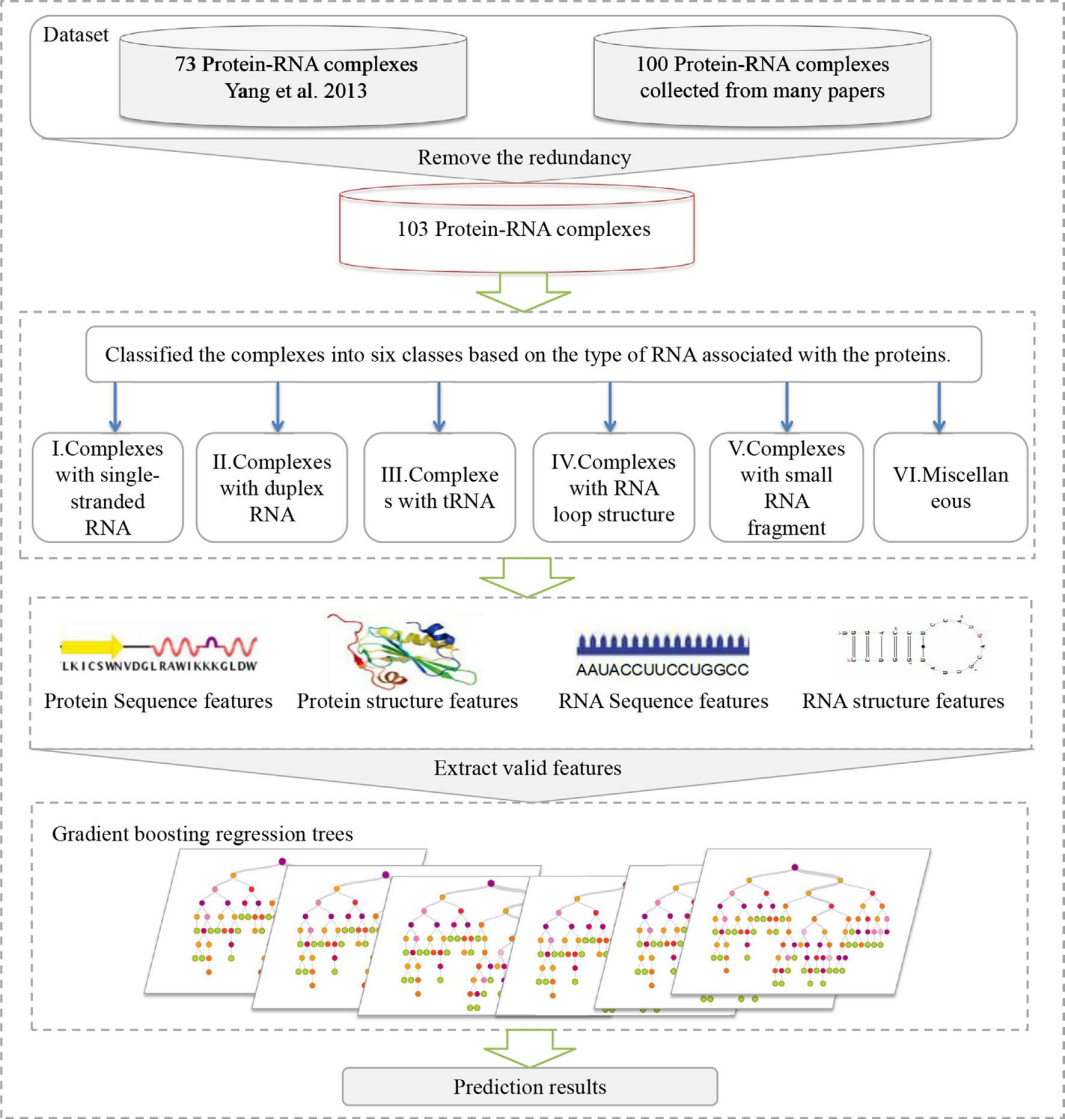
The flowchart of the PredPRBA method for predicting the binding affinity of protein-RNA complexes. It involves four steps: **(A)** collection of complexes with experimentally measured binding affinities from publications. **(B)** Classification of complexes according to the type of RNAs interacting with proteins. **(C)** Feature extraction from sequence and structure from proteins and RNAs for building a predictive model. **(D)** Training gradient boosting regression tree models.

In this work, we have developed a method, referred to as PredPRBA, to predict the quantitative binding affinity of protein-RNA complexes. The flowchart of our method is shown in **Figure 1**. We classified the protein-RNA complexes into six categories based on the type of RNA interacting with proteins Bahadur et al. (2008), and set up gradient boosted regression trees (GBRT) (Temel et al., 2014) models for predicting the binding affinity of each class of complexes. For each class of protein-RNA complexes, we have conducted systematic analysis on the importance of features in predicting the binding affinity and found that the structural features play a vital role in governing protein-RNA binding affinity. Our method showed correlation coefficients ranging from 0.723 to 0.897 on leave-one-out cross-validations. We have conducted a performance comparison of our method with several typical regression methods and an existing binding affinity predictive method, the empirical experiments have illustrated that our method achieved the best performance. To our knowledge, the dataset of quantitative binding affinity of protein-RNA complexes we built is the largest one to date. Also, PredPRBA is the first devoted to the prediction of quantitative protein-RNA binding affinity. In addition, a user-friendly web server has been developed to predict the binding affinity of protein-RNA complexes.

## Materials and Methods

### Dataset

We primarily collect 173 protein-RNA complexes to extract quantitative protein-RNA binding affinity, among which 73 complexes come from a non-redundant protein-RNA binding benchmark dataset (Yang et al., 2013), and other 100 complexes are collected from relevant publications. In particular, all the complexes meet the criteria: 1) the interacting partners are proteins and RNAs, 2) absolute value of binding affinity is known, 3) The complexes containing protein chains with 30 or more amino acid residues and RNA chains with 2 or more nucleotides were retained. To reduce the redundancy, we remove the complexes with protein sequence similarity greater than 40% using the CD-HIT (Li, 2015), which can cluster the proteins by sequence similarities and select a representative one for each cluster. As a result, we obtain 103 non-redundant protein-RNA complexes, and build a data set of protein-RNA binding affinities (listed in **Supplementary Table 1**), along with experimental situations (pH value and temperature). We defined dissociation Gibbs free energy Δ*G* as the binding affinity according to the definition of protein-RNA binding affinity proposed by Yang et al. study (Yang et al., 2013). Moreover, the Δ*G* is calculated by the equation as below:

(1) $$∆G=-RT\ln\;K_{d}$$

Where *K_d_* is the dissociation constant, *R* is the gas constant (1.987 × 10^-3^kcal mol^-1^K^-1^), and *T* is the temperature. It can be seen that the binding affinity is a real-valued quantity.

### Classification of Complexes

It is worth noting that previous findings have demonstrated that the structure of RNA molecules greatly influences the binding affinity between proteins and RNAs (Li et al., 2012), namely the binding affinities regarding different type of RNAs depend on different features related to RNA structure. In fact, the classification of protein-RNA complexes, according to RNA types, has been adopted in the previous study for building prediction models (Bahadur et al., 2008). Therefore, we divide the protein-RNA complexes into six groups according to the Nucleic Acid Database (NDB) (Coimbatore Narayanan et al., 2013): I) complexes with single-stranded RNA, II) complexes with duplex RNA, III) complexes with tRNA, IV) complexes with RNA loop structure, V) complexes with small RNA fragment, VI) miscellaneous complexes.

### Features Extraction

We extract a total of 37 kinds of features to predict the binding affinity of the protein-RNA complexes. These features can be mainly separated into four categories, including features based on protein sequences and protein structures, features based on RNA sequences and RNA structures.

#### Protein Sequence-Based Features

We extract the protein sequences from the PDB files and then calculate the total molecular mass of the protein fraction based on the molecular weight of each amino acid. Also, the total number of hydrogen bonds (McDonald and Thornton, 1994) contained in the protein-RNA complexes was calculated based on the number of hydrogen bonds held in each amino acid. Moreover, we calculate the number of hydrophilic and hydrophobic residues (Andersen et al., 1999) in the proteins, the percentage of hydrophilic residues in the protein, the percentage of hydrophobic residues in the protein, the number of the aromatic and positively charged residues and the percentage of aromatic and positively charged residues (Monaco-Malbet et al., 2000) in the proteins, the number of the charged residues in protein, the percentage of the charged residues in protein, the number of the polar residues in protein, the percentage of the polar residues in protein.

#### Protein Structure-Based Features

We use the DSSP algorithm (Kabsch and Sander, 1983) to obtain the secondary structure information of the interacting proteins. We obtained the secondary structure information, including the number of *α*-helix and *β*-sheet, the molecular weight of *α*-helix (Qian and Sejnowski, 1988; Chakrabarti and Janin, 2002) and *β*-sheet (Albeck and Schreiber, 1999), the percentage of α-helix and *β*-sheet in proteins. Meanwhile, we sum the solvent-accessible surface area obtained from the protein amino acids in each complex to obtain the total value of the relative solvent accessible surface area (RASA) (Xia et al., 2010).

#### RNA Sequence-Based Features

We use the RNA sequences in the protein-RNA complexes to obtain the molecular mass of the RNA molecules. The computational formula is as below.

(2) $$W_{RNA}=329.2*A+306.2*U+305.2*C+345.2*G+159$$

in which *A*, *G*, *C*, *U* represent the numbers of four types of bases in the RNA sequence, respectively.

#### RNA Structure-Based Features

A number of features based on the RNA structure are derived to predict protein-RNA binding affinities. We use the RNA fold in ViennaRNA (Lorenz et al., 2011) to predict the frequency of the MFE structure and ensemble diversity. Also, the features of cWW (Cis Watson-Crick/Watson-Crick) (Leontis and Westhof, 2001) and Base-Phosphate (Stombaugh et al., 2009) are predicted. We use the RNAVIEW tool (Bahadur et al., 2008) to get four features, including the number of cWW and the relative frequency of cWW and the number of 0BPh in Base-Phosphate and the relative frequency of 0BPh.

### Prediction Model and Validation

#### GBRT Algorithm

Ensemble learning algorithms are a family of powerful machine-learning techniques that have shown considerable success many applications (Caruana and Niculescu-Mizil, 2005; Tang et al., 2017; Kuang et al., 2018; Li et al., 2018; Pan et al., 2018; Wang et al., 2018; Zheng et al., 2019). We chose a boosting ensemble model, the gradient boosted regression trees (GBRT) algorithm, to build the prediction model for protein-RNA binding affinity, thanks to its ability to handle different types of data and strong predictive power. Precisely, GBRT is an iterative regression decision tree algorithm composed of multiple regression trees, and the predictions of all the trees are taken into account to get the final decision.

Without loss of generality, the features and the real-valued binding affinities can be described as an *n*-dimension vector. Let us denote the features by *x* = (*x*_1_, *x*_2_, …, *x_n_*) where *x_i_* ∈ ***R*** and the corresponding binding affinity by *y*. The goal of predicting binding affinity real value of the protein-RNA complexes is to find a function F*(*x*) that maps *x* to *y*, such that over the joint distribution of all (*y*, *x*)-values, the expected value of some specified loss function Ψ(*y*, *F* (*x*)) is minimized as follows:

(3) $$\;\begin{array}{l} F^{*}(x)=\underset{F(x)}{\arg\;\min}\;E_{y,x}\Psi \left(y,F(x)\right)\\ =\underset{F(x)}{\arg\;\min}\;E_{x}[E_{y}(\Psi \left(y,F\left(x\right)\right)|x] \end{array}$$

Let ${\left\{y_{i},x_{i}\right\}}_{1}^{N}$ be a set of training data, *N* is the number of samples in the training set. The GBRT algorithm iteratively constructs *M* different weak learners *h*(*x*, *Θ*_1_), …, *h*(*x*, Θ*_M_*) which consist of regression trees of fixed size from training set and constructs the following additive function *F*(*x*):

(4) $$F\left(x\right)=\beta_{0}+\overset{M}{\underset{m=1}{\sum }}\beta_{m}h\left(x,\Theta_{m}\right)$$

where *β_m_* and *Θ_m_* are a weight and vector of parameters for the *m*-th weak regression tree *h*(*x*, *Θ_m_*), respectively, and *β*_0_ is an initial constant. Both the weight *β_m_* and the parameters *Θ_m_* are iteratively determined from weak learner 1 to *M* so that the loss function Ψ(*y*, *F*(*x*)) is minimized. Formally, *β_m_* and *Θ_m_* for the *m*-th regression tree are determined as follows:

(5) $$\;\left(\beta_{m},\Theta_{m}\right)=\underset{\beta ,\Theta }{\arg\;\min}\;\overset{N}{\underset{i=1}{\sum }}\Psi \left(y_{i},F_{m-1}(x_{i})+\beta h\left(x_{i},\Theta \right)\right)$$

where *F_m_*_-1_(*x*) is the (*m*-1)th additive function combined from the first to the (*m*-1)th weak regression tree.

However, it is not straightforward to solve Eq. (5). Therefore, GBRT separately and approximately estimates (ǀ*β_m_*, *Θ_m_*) in a simple two-step fashion (Friedman, 2001). For the estimation of the parameters *Θ_m_*, we determine them so that the function defined by the regression tree approximates a gradient with respect to the current function F*_m_*_-1_(*x*) in the sense of least-square error as follows:

(6) $$\Theta_{m}=\underset{\Theta }{\arg\;\min}\;\overset{N}{\underset{i=1}{\sum }}{\left({\tilde{y}}_{im}-h\left(x_{i},\Theta \right)\right)}^{2}$$

where ${\tilde{y}}_{im}$ is the gradient and is given by

(7) $${\tilde{y}}_{im}=-{\left[\frac{\partial \Psi \left(y_{i},F\left(x_{i}\right)\right)}{\partial F\left(x_{i}\right)}\right]}_{F\left(x\right)=F_{m-1}\left(x\right)}$$

When the *m*-th regression tree using the Θ*_m_* has *L_m_* leaf nodes, the regression tree is given by

(8) $$\begin{array}{l} h(x,{\left\{R_{lm}\right\}}_{l=1}^{L_{m}})=\overset{L_{m}}{\underset{l=1}{\sum }}{\tilde{y}}_{lm}l\left(x\in R_{lm}\right) \end{array}$$

where *R_lm_* is a disjoint region that the *l*th leaf node of the *m*-th regression tree defines. *l*(). is a Boolean function that outputs 1 in case the argument of the function is true. ${\tilde{y}}_{lm}$ is a constant for the *R_lm_* th region, defined as the mean of training data that belongs to the *l*th leaf node of the *m*-th regression tree. The weight *β_m_* can be straightforwardly chosen using line search:

(9) $$\beta_{m}=\underset{\beta }{\arg\;\min}\;\overset{N}{\underset{i=1}{\sum }}\Psi \left(y_{i},F_{m-1}(x_{i})-\beta \frac{\partial \Psi (y_{i^{,F_{m-1}\left(x_{i}\right)}})}{\partial F_{m-1}\left(x_{i}\right)}\right)$$

Then, a new additive function *F_m_*(*x*) is updated as follows:

(10) $$F_{m}(x)=F_{m-1}(x)+\nu \overset{L_{m}}{\underset{l=1}{\sum }}\beta_{m}{\tilde{y}}_{lm}l(x\in R_{lm})$$

where 0 < *v* < 1 is a shrinkage parameter, also called the learning rate, to scale the step length the gradient descent procedure. Finally, the resulting binding affinity value *y* corresponding to the features *x* is given by: *y* = *F_M_*(*x*).

#### Performance Measures

The performance is evaluated using the Pearson correlation coefficient (Kader and Franklin, 2008) between the predicted binding affinities and real values. The Pearson correlation coefficient *r* is defined as the linear correlation between two random variables *X* and *Y*:

(11) $$r=\frac{\sum_{i=1}^{n}(x_{i}-\bar{x})\left(y_{i}-\bar{y}\right)}{\sqrt{\sum_{i=1}^{n}{\left(x_{i}-\bar{x}\right)}^{2}}\sqrt{\sum_{i=1}^{n}{\left(y_{i}-\bar{y}\right)}^{2}}}$$

in which *n* is the sample size, *x_i_*, *y_i_* are the single samples indexed with *i*, and $\bar{x}$ and $\bar{y}$ are the sample means, i.e. $\bar{x}=\frac{1}{n}\overset{n}{\underset{i=1}{\sum }}x_{i}$ and $\bar{y}=\frac{1}{n}\overset{n}{\underset{i=1}{\sum }}y_{i}$.

In addition, the average absolute error(MAE) (Willmott and Matsuura, 2005) is the average of the absolute values of the deviations of all individual samples from the arithmetic average. It can better reflect the actual situation of the prediction error. The coefficient of determination (R2) (Miller et al., 2006) can measure whether the future sample is likely to be well predicted by the model, with a score of 1 indicating the best effect.

#### Features Selection

We independently conduct iterative feature selection for each class of protein-RNA complexes, as the binding affinity of the different class of complexes is influenced by the structure of RNAs and proteins. In particular, we build the protein-RNA binding affinity prediction models iteratively using each feature and compute the performance measure Pearson correlation coefficient. Next, we sort the features in descending order according to the correlation coefficient and select the top 10 features for each class complex. Finally, we adopt the greedy algorithm to add one feature to the optimal feature set at each step until the performance stops to increase. The selected features are shown in the **Table 1** for each class of protein-RNA complexes. Overall, the numbers of features included in the final optimal feature set are no more than 6 for all six classes of complexes.

**Table 1:** Selected features to predict protein-RNA binding affinity of each class of protein-RNA complexes.

	Class I	Class II	Class III	Class IV	Class V	Class VI
molecular weight of RNA	√					
total value of the relative solvent accessible surface area				√	√	
number of hydrophilic residues in the protein			√		√	
number of hydrophobic residues in the protein		√				
% of hydrophilic residues in the protein						√
% of hydrophobic residues in the protein			√	√	√	√
% of the aromatic and positively charged residues in the protein				√		
number of the aromatic and positively charged residues in the protein						√
number of the charged residues in protein			√		√	
number of the polar residues in protein		√		√		
molecular weight of *α*-helix			√			√
molecular weight of *β*-sheet					√	
number of cWW	√					
relative frequency of cWW	√	√		√		
frequency of the MFE structure	√					

## Results

### Significance of Protein-RNA Complex Classifications

We first conduct an experiment to check the significance of the classification of protein-RNA complexes based on RNA types. For each class of complexes, we use the top 1 and 2 features to train GBRT prediction models and compute the performance measures, respectively. As a contrast, we take all the complexes as a whole to train the prediction model using the top 1 and top 2 features. The results are shown in **Table 2**, it can be found that the prediction accuracy after classification is much better than that of before classification of complexes. For the prediction models built on top 1 features, the correlation coefficients are more than 0.5 in half of the six classes of complexes, whereas the whole set of complexes get only 0.178 correlation coefficient. In fact, the best correlation coefficient before the classification we can obtain is less only 0.48 using optimal feature set (not shown in the table). We think the reason lies in that different class of complexes have very weak relevance, which leads to the difficulty of modeling. For example, the number of hydrophobic residues in the protein has a positive impact on the complex that binds duplex RNA but causes a decrease in the correlation coefficient of the complex that binds the single-stranded RNA. Therefore, we highlight the significance of protein-RNA complex classifications before building practical prediction models.

**Table 2:** Performance of models built on the best one and two features for six classes of protein-RNA complexes.

	Number of complexes	Maximum correlation coefficient(r)	
		Single property	Two properties
Class I	21	0.565	0.725
Class II	34	0.452	0.546
Class III	8	0.567	0.669
Class IV	9	0.616	0.663
Class V	11	0.422	0.521
Class VI	20	0.511	0.615
All	103	0.178	0.332

### Prediction of Binding Affinity

**Figure 2:**
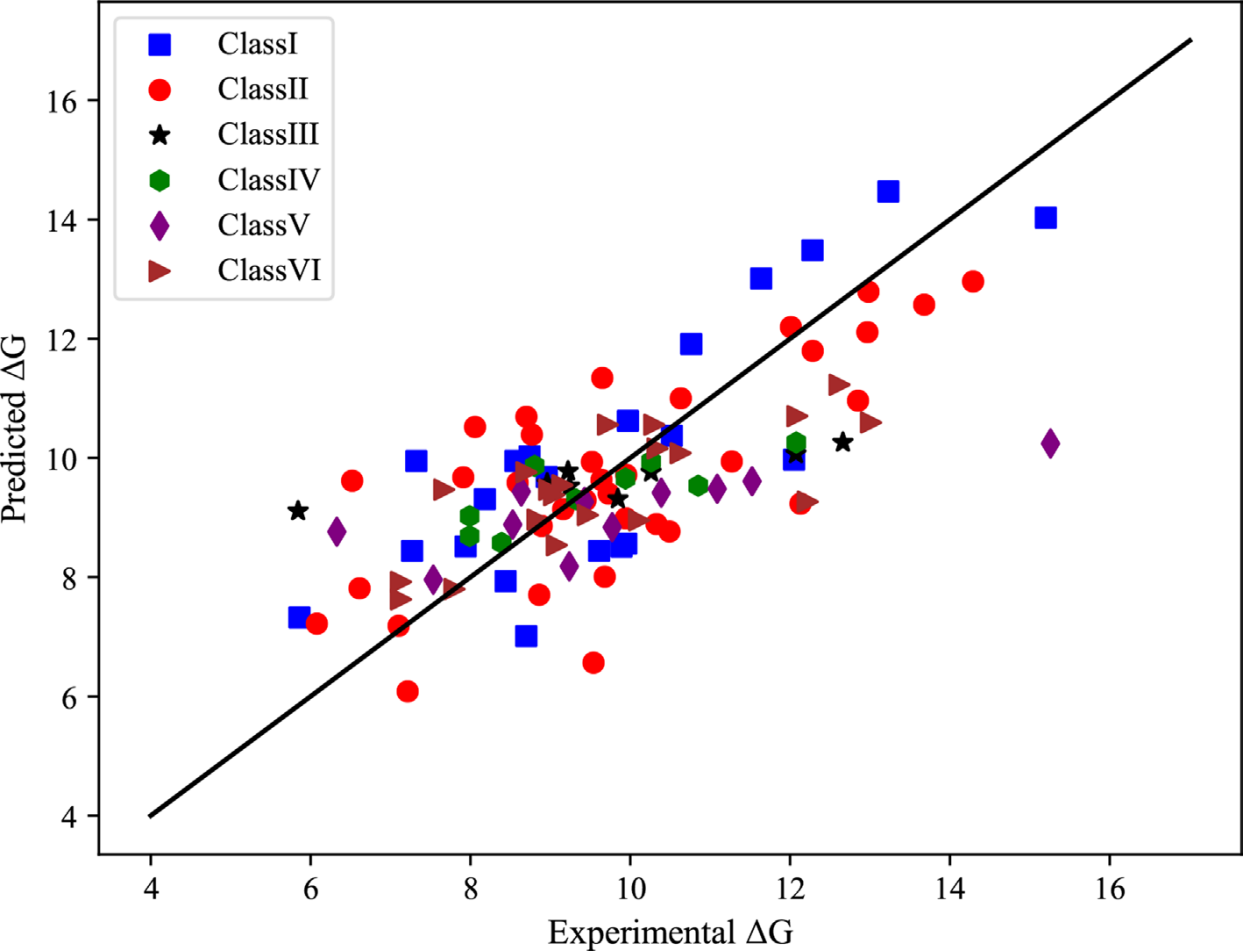
Scatterplot in the coordinate of experimental *vs* predicted binding affinities of six classes of protein-RNA complexes.

For each class of protein-RNA complexes, we train the GBRT model using the selected features to predict binding affinities. The correlation coefficients, together with MAE and R2 measures, are shown in **Table 3**. We notice that the correlation coefficients are more than 0.73 for all complexes classes, indicating that the predicted binding affinities are strongly related to real values. Also, we show the scatter plot in the coordinate of experimental *vs* predicted Δ*G* in **Figure 2**, from which we can find that most points are located close to the diagonal line.

**Table 3:** Performance measures of Pred PRBA on leave-one-outcrossvalidations.

	Correlation coefficient(r)	Mean absolute error(MAE)	Coefficient of determination(R2)
Class I	0.818	1.215	0.623
Class II	0.731	1.145	0.518
Class III	0.894	1.270	0.288
Class IV	0.803	0.749	0.489
Class V	0.768	1.425	0.255
Class VI	0.762	0.879	0.531
Average value	0.796	1.114	0.451

**Figure 3:**
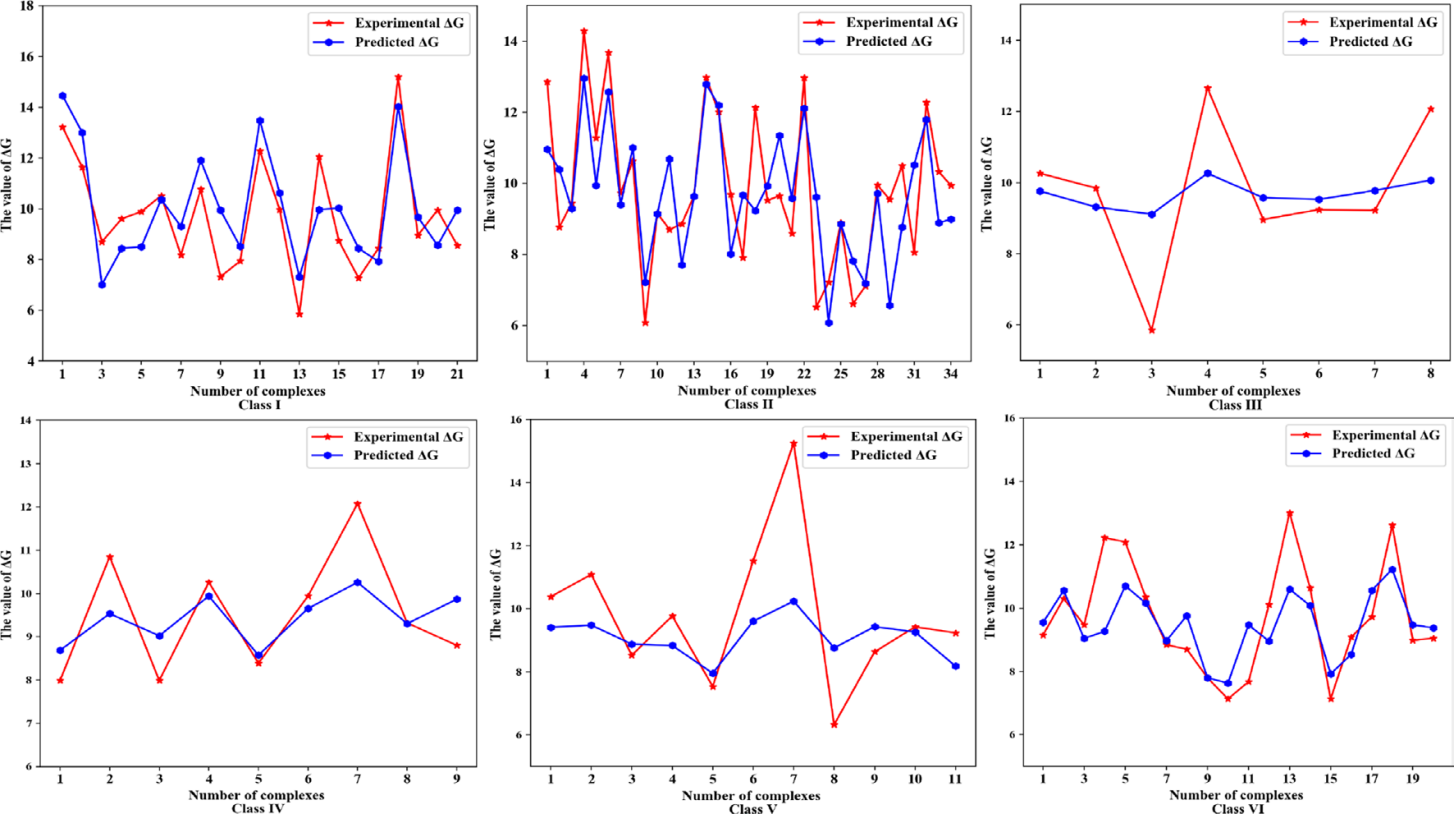
The predicted and actual binding affinities, represented by Δ*G*, of each protein-RNA complex in six classes of complexes.

Next, we further evaluate the performance of the method for predicting the binding affinity in different classes and reveal the features that dominate the prediction of binding affinity of protein-RNA complexes. The predicted and actual values of binding affinities for each complex in six classes of complexes are shown in **Figure 3**, respectively.

#### Complexes With Single-Stranded RNA

In this class of complex, proteins interact with single-stranded RNA molecules that are very common *in vivo*. There are 21 protein-RNA complexes in this class, and the binding affinity has the variation of 10 kcal mol^-1^, with the lowest value being 5.86 kcal mol^-1^ and the highest value of 15.2 kcal mol^-1^. Our model built on four types of features has achieved the correlation coefficient of 0.818 by leave-one-out cross-validations. As shown in **Table 1**, we can see that the features based on RNA sequence and structure, especially the molecular weight of RNA and the frequency of the MFE structure, play the dominant role in predicting the binding affinity of this class of complexes. In addition, the number and the relative frequency of cWW are also significant factors for predicting the binding affinity of complexes associated with single-stranded RNA. These RNA-related features indicate that RNA molecules play a major role in interacting with proteins in this class of complexes.

#### Complexes With Duplex RNA

The interacting partners in this class of protein-RNA complexes are protein and double-stranded RNA. The binding affinities follow the range of 6–14 kcal mol^-1^. Three selected features are used to build the prediction model that obtain the correlation coefficient 0.731. The physicochemical properties of the protein fraction play most important role in the prediction of the binding affinity of this class of complexes. In particular, the number of hydrophobic residues in the protein and the number of the polar residues in proteins are also features of importance, which demonstrate that the physicochemical properties of the interacting proteins have a major impact on the interaction between proteins and double-stranded RNA.

#### Complexes Wth tRNA

This class of complexes is composed of proteins and tRNA molecules, and four features enable our model to achieve a correlation coefficient of 0.872. From **Table 1**, we find that the four selected types of features are all related to proteins. The physicochemical properties of the proteins are critical to predicting the binding affinities, including the number of hydrophobic residues, the percentage of hydrophobic residues and the number of the charged residues in the interacting proteins. Among the structural features of proteins, the molecular weight of the α-helix also plays an important role in predicting the binding affinity. These indicate that the interacting proteins mainly determine the binding affinity of the complexes with tRNA.

#### Complexes With RNA Loop Structure

RNA loop structure includes many types, such as hairpin loops, internal loops, etc. (Bahadur et al., 2008). Our prediction model, based on five features, can obtain a high correlation coefficient of 0.803. Among 37 features, the protein-related features play a major role in predicting the binding affinity of complexes with loop-structure RNAs. The physicochemical properties of proteins still play an important role, including the percentage of hydrophobic residues, the percentage of the aromatic and positively charged residues and the number of the polar residues in the protein, are the top three dominant features. Meanwhile, the secondary structural features of proteins and RNAs, including the total value of the relative solvent accessible surface area and the relative frequency of cWW, are also two essential factors in predicting the binding affinity of this type of complex. The structural features of RNA also play a key role in the prediction of the binding affinity of the complex.

#### Complexes With Small RNA Fragment

One interacting partner of this class of protein-RNA complexes is the small RNA fragment. There are 11 complexes in our dataset, and the average binding affinity is 9.78 kcal mol^-1^. As shown in **Table 1**, we see that all selected features for this class of complexes are extracted from proteins. Among the protein sequence-based features, the physicochemical properties play the most important role, including the number of hydrophilic residues, the percentage of hydrophobic residues and the number of the charged residues in the protein. Among the protein structure-based features, the total value of the relative solvent accessible surface area and the molecular weight of *β*-sheet have an essential function in the interaction between proteins and small RNAs.

#### Miscellaneous Complexes

The complexes that do not fall into the above five categories are assigned to miscellaneous. The reason is that the structure of RNA in this class of complexes is uncertain and software available cannot determine their specific structures, we thereby assumed that the features influencing the binding affinity of this class of complexes might be different from other classes. This class consists of 20 complexes, and the binding affinities range from 6 to 15 kcal mol^-1^. The set of four features are included in our model to predict the binding affinity, and the correlation coefficient is 0.76 on leave-one-out cross-validations. The molecular weight of α-helix and the number of the aromatic and positively charged residues in the protein are identified as important factors influencing the binding affinity. Moreover, among the protein sequence-based features, the percentage of hydrophilic and hydrophobic residues in the protein also play a vital role.

### Utilization of Both Protein-Based and RNA-Based Features Improve Performance

To verify that the utilization of both protein-derived features and RNA-derived features improve the performance of our prediction models, we build other two GBRT prediction models, referred to as protein-based and RNA-based prediction models, using only protein-derived features or RNA-derived features alone. Next, we compare their performance to that of PredPRBA that takes advantage of both protein-derived features and RNA-derived features. **Table 4** shows the performance of three prediction models on six classes of complexes. We find that the models using only features derived from proteins or RNAs achieve fairly good performance for some classes of protein-RNA complexes, while utilization of the features derived from both proteins and RNAs yields to the best performance.

**Table 4:** Performance comparison of PredPRBA to protein-based and RNA-based prediction models.

	Protein-based model	RNA-based model	PredPRBA
Class I	0.562	0.818	**0.818**
Class II	0.652	0.436	**0.731**
Class III	0.894	0.634	**0.894**
Class IV	0.642	0.621	**0.803**
Class V	0.768	0.547	**0.768**
Class VI	0.762	0.635	**0.762**
Average	0.71	0.62	**0.80**

### Performance Comparison to Sequence Feature-Based and Structure Feature-Based Models

Inspired by the study of protein-RNA interactions by Liu et al. (Liu and Miao, 2016), we compare the performance of PredPRBA to the models built on sequence feature-based or structure feature-based alone. In particular, we use only 20 sequence-based features extracted from protein and RNA sequences to train the sequence feature-based GBRT prediction model, and use only 17 structure-based features from proteins and RNAs to build the structure feature-based GBRT prediction models for each class of protein-RNA complexes, respectively. **Table 5** shows the performance measures of PredPRBA, the sequence feature-based models, and structure feature-based models. It can be seen that sequence feature-based and structure feature-based models also achieve fairly good performance on all six classes of protein-RNA complexes, while PredPRBA performs even better by virtue of the inclusion of both structural features and sequence features.

**Table 5:** Performance comparison of PredPRBA to sequence feature-based and structur efeature-based models.

	Sequence-based model	Structure-based model	PredPRBA
Class I	0.661	0.711	**0.818**
Class II	0.618	0.635	**0.731**
Class III	0.883	0.765	**0.894**
Class IV	0.696	0.735	**0.803**
Class V	0.661	0.697	**0.768**
Class VI	0.736	0.665	**0.762**
Average	0.71	0.70	**0.80**

### Performance Comparison With Typical Regression Methods

**Figure 4:**
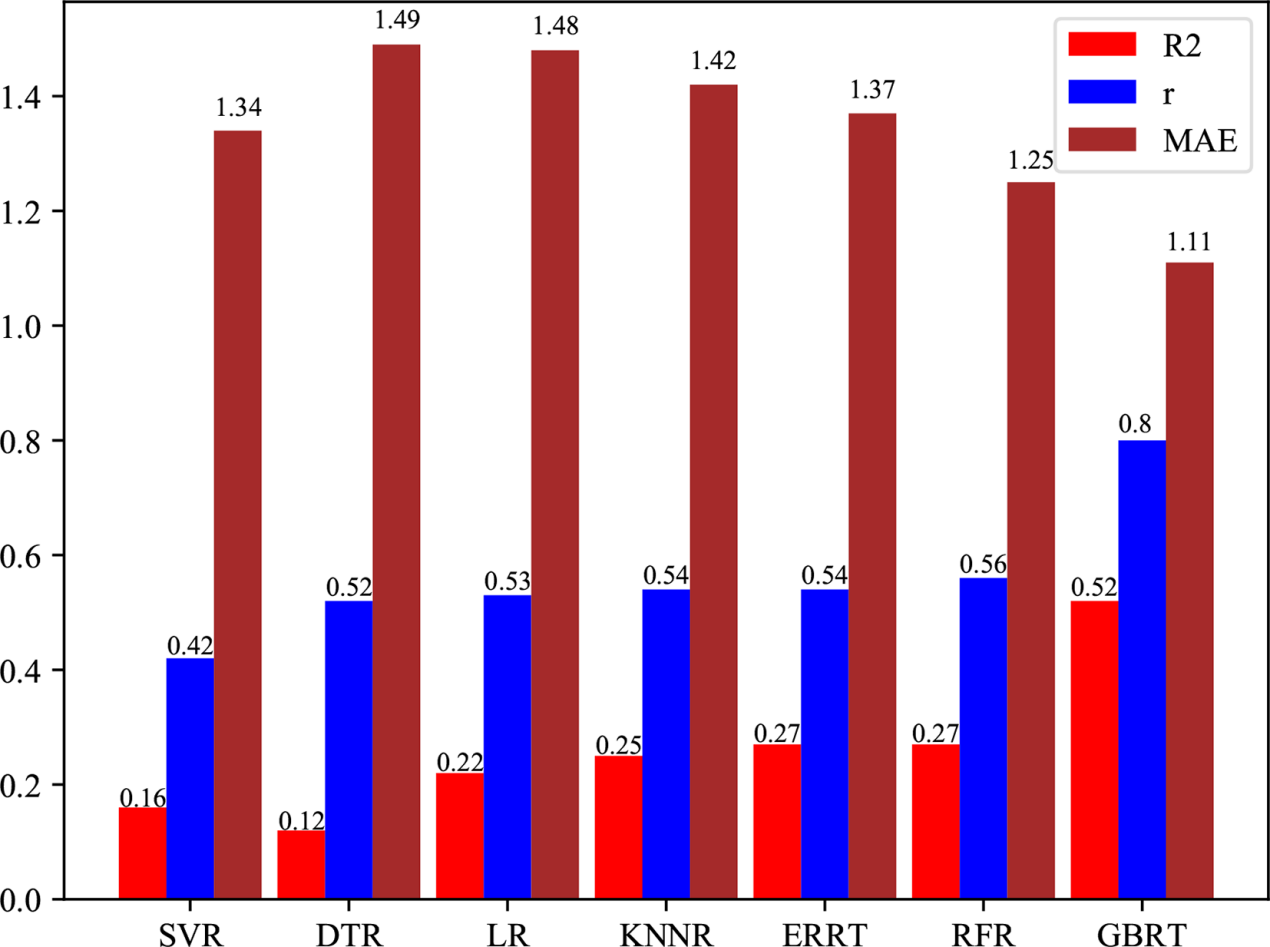
Comparison of mean correlation coefficients over six classes of protein-RNA complexes between PredPRBA and typical regression methods.

We evaluate PredPRBA by conducting performance comparison with several other typical regression methods, such as Linear Regression (LR) (Jammalamadaka, 2003), K-nearest Neighbor Regression (KNNR) (Kramer, 2011; Kuang et al., 2019), SVM Regression (SVR) (Cherkassky and Ma, 2004), Decision Tree Regression (DTR) (Xu et al., 2005), Random Forest Regression (RFR) (Biau and Devroye, 2010) and Extremely Randomized Regression Trees (ERRT) (Geurts and Louppe, 2011). As shown in **Table 6**, we find that PredPRBA performs significantly better than other regression methods for all classes of complexes. Furthermore, **Figure 4** shows the mean values of the performance measures, including correlation coefficients, MAE and R2 values, for different regression methods over six classes of complexes. For instance, the average correlation coefficient of PredPRBA achieves 0.80, which is much greater than other methods. Accordingly, we can see that by the PredPRBA model has the least mean MAE value, as well as the largest mean R2 value. The experimental results show that the GBRT algorithm empowers better performance to our method than other regression methods.

**Table 6:** Comparison of correlation coefficients between PredPRBA and other regression algorithms.

	SVR	DTR	LR	KNNR	ERRT	RFR	PredPRBA
Class I	0.541	0.356	0.604	0.411	0.760	0.641	**0.818**
Class II	0.356	0.621	0.456	0.476	0.685	0.695	**0.731**
Class III	0.708	0.449	0.634	0.628	0.458	0.535	**0.894**
Class IV	0.389	0.669	0.696	0.602	0.588	0.724	**0.803**
Class V	0.366	0.395	0.432	0.492	0.215	0.343	**0.768**
Class VI	0.157	0.377	0.374	0.636	0.519	0.400	**0.762**
Average	0.42	0.52	0.53	0.54	0.54	0.56	**0.80**

### Performance Comparison With Existing Approach

The SPOT-Seq-RNA (Yang et al., 2014) is another method for predicting binding affinity. It is worth noting that there are quite a few existing methods developed to predict protein-protein binding affinity, but these methods cannot be applicable for the prediction of protein-RNA binding affinity, as they do not take the RNA-related features into account. Therefore, we include only SPOT-Seq-RNA for performance comparison and run this method to predict the binding affinity of the complexes in our dataset. **Table 7** shows a comparison of the correlation coefficients of PredPRBA and SPOT-Seq-RNA, from which we can see that our approach greatly outperforms SPOT-Seq-RNA. In fact, the performance of SPOT-Seq-RNA is not steady over the six classes of protein-RNA complexes, i.e., it obtains fairly good performance on class I and V complexes, but performs poor on other classes of complexes.

**Table 7:** Comparison of correlation coefficients between SPOT-Seq-RNA method and Pred PRBA.

	Number of complexes	Correlation coefficient(r)	
		SPOT-Seq-RNA	PredPRBA
Class I	21	0.442	0.818
Class II	34	-0.044	0.731
Class III	8	-0.038	0.894
Class IV	9	0.172	0.803
Class V	11	0.756	0.768
Class VI	20	0.386	0.762
Average	17	0.276	0.796

## Conclusion

In this paper, we propose a method for predicting the binding affinities of protein-RNA complexes using the sequence-based and structure-based features. As far as our knowledge, the data set of binding affinities of 103 protein-RNA complexes we built is the largest dataset to date. For each class of protein-RNA complexes, we have conducted systematic analysis on the importance of features in predicting the binding affinity and found that the structural features play a vital role in governing protein-RNA binding affinity. We also compared our method with several typical regression methods and the existing binding affinity predictive method, and the performance comparison has verified that our method achieved the best performance. In addition, we have also developed a web server for predicting the binding affinity of protein-RNA complexes, which is free and open to the academic community.

## Data Availability

The datasets for this study can be found in the http://PredPRBA.denglab.org/.

## Author Contributions

LD, WY, and HL designed the study and conducted experiments. LD and WY performed statistical analyses. LD and HL drafted the manuscript. WY prepared the experimental materials and benchmarks. All authors have read and approved the final manuscript.

## Funding

This work was supported by the National Natural Science Foundation of China under grant no. 61672541 and no. 61672113, and Natural Science Foundation of Hunan Province under grant no. 2017JJ3412.

## Conflict of Interest Statement

The authors declare that the research was conducted in the absence of any commercial or financial relationships that could be construed as a potential conflict of interest.
